# Assessment of Dental Anxiety and Hemodynamic Changes during Different Dental Procedures: A Report from Eastern Saudi Arabia

**DOI:** 10.1055/s-0041-1740222

**Published:** 2022-01-06

**Authors:** Zainab Alghareeb, Kawther Alhaji, Bayan Alhaddad, Balgis Gaffar

**Affiliations:** 1 Intership Program, College of Dentistry, Imam Abdulrahman bin Faisal University, Dammam, Saudi Arabia; 2 Department of Biomedical Dental Sciences, College of Dentistry, Imam Abdulrahman bin Faisal University, Dammam, Saudi Arabia; 3 Preventive Dental Sciences Department, College of Dentistry, Imam Abdulrahman bin Faisal University, Dammam, Saudi Arabia

**Keywords:** dental anxiety, modified dental anxiety scale, hemodynamic changes, blood pressure, local anesthetic, dental treatment

## Abstract

**Objectives** This study aimed to investigate hemodynamic changes in healthy adult patients during different dental procedures and evaluate whether these changes were associated with patients' dental anxiety.

**Materials and Methods** A convenience sample of 119 patients of both genders undergoing routine dental care participated in the study. Participants responded to the Arabic version of the modified dental anxiety scale (MDAS) and a self-structured questionnaire. Each patient had their blood pressure, heart rate, and oxygen saturation measured at three points: before, during, and after the dental procedure using an electronic sphygmomanometer. MDAS scores were categorized into no anxiety, mild, moderate or severe anxiety, while readings of heart rate and blood pressure were categorized into no change, increased or decreased and either “no change” or “increased” for oxygen saturation. Chi-square test was used to investigate the association between the study variables and a *p* value of < 0.05 was considered statistically significant. SPSS version 20 was used in the analysis.

**Results** Mean ( ± standard deviation [SD]) of MDAS was 11.12 ( ±  3.9) an indicative of moderate dental anxiety. No changes in blood pressure, heart rate, or in oxygen saturation were observed on 39.5%, 54.6% and 97.5% among the study participants, respectively. Half of the participants avoided dental care, with dental anxiety being the main reason for that (26.1%). Pattern of dental visits was significantly associated with MDAS scores ( *p* = 0.042). There were significant changes in blood pressure ( *p* = 0.0003), heart rate ( *p* = 0.01) but not in oxygen saturation ( *p* = 0.33). Changes in blood pressure, heart rate, and oxygen saturation were not associated with dental anxiety *p* = 0.15, 0.10, and 0.99, respectively.

**Conclusion** The results of this study indicate that the type of dental procedure may cause dental anxiety and cause hemodynamic changes. Therefore, close monitoring of patients with dental anxiety during the treatment is advised.

## Introduction

Dental fear, anxiety, and phobia terms have been used in the dental literature for decades, however, without being clearly demarcated. 1 Dental anxiety can be defined as vague unpleasant feeling or nervousness from receiving dental care. 1 2 3 While dental fear is the feeling associated with the sense of threat. 1 2 On the other hand, avoidance response of the perceived danger can be known as phobia, which usually leads to unrealistic and intense fear of a specific stimulus. 1 4 Dental anxiety can be associated with certain factors such as traumatic dental experience, fear of pain or unknown, and environmental factors such as the smell or noise of dental materials and instruments. 3 5 Anxious patients are usually less cooperative and experience more pain 3 , thereby affecting the quality of care provided as well as the overall treatment outcome. 6 7 8 It is well-established that avoiding dental visits for very long time can be the consequence of high levels of dental anxiety. 2 The extent of dental anxiety expressed in adulthood is thought to originate mainly from previous traumatic experience in childhood. 9

The provision of dental care is usually associated with higher anxiety levels compared with other medical health fields. 8 Fifty to eighty percent of adults in United States experience degree of dental anxiety ranging from mild to severe. 10 Local estimates of dental anxiety varied across the different major cities of Saudi Arabia, 5 6 7 11 ranging from 27% in Dammam 11 up to 54.5% in Riyadh. 7

Type of dental treatment was found to affect the amount of anxiety that the patient experiences 12 regardless of age. 13 Studies showed that vital signs changes had a positive correlation with moderate-to-severe dental anxiety. 14 As reported by Al-Faleg, extractions and root canal treatments were the most anxiety-provoking procedures to individuals in Buraydah, Saudi Arabia. 5 Gaffar and colleagues found that anesthetic injections followed by surgical procedures were the most dreadful dental procedures among sampled adults in Dammam. 11 On the other hand, Gadve et al reported an obvious increase in heart rate and diastolic blood pressure during surgical procedures in patients with high dental anxiety, such as during ostectomy and extraction of the teeth. 8 Similarly, Patini et al observed increased pain perception and heart rate with administration of local anesthesia to children using the traditional syringe compared with a computer-controlled device 13

Although a wealth of studies investigated the prevalence and causes of dental anxiety among different age groups 1 2 3 4 5 6 7 9 10 11 12 however, few investigated the impact of different dental procedures on vital signs and dental anxiety 8 13 14 15 and none of these were conducted in Eastern societies. Dental anxiety is affected by sociodemographic, cultural, 2 as well as behavioral factors such as pain perception; 16 therefore, how individuals respond and express it is expected to vary across countries. Such information is crucial to provide better understanding about the dental procedures that increase dental anxiety, thereby achieving better treatment outcome and communication with anxious patients and preventing vasovagal syncope by monitoring the vital signs of anxious patients. Therefore, the purpose of this study was to investigate the association between dental anxiety and changes in vital signs during different dental procedures.

## Materials and Methods

This was a cross sectional, hospital-based study conducted during the period from January to July 2019. Ethical approval was obtained from the Deanship of Scientific Research—Imam Abdulrahman Bin Faisal University, Dammam (IRB-2019–02–080).

The sample size was calculated considering the percentage of extremely patients anxious in the waiting area (49%) and during the injection of dental anesthesia (64%). 17 The sample size estimation based on the comparison of two proportions (0.49 and 0.64) using the World Health Organization (WHO) sample size calculator, 5% margin of error, 80% power and 95% confidence interval (CI) was 135 cases, which were to be evaluated for dental anxiety and expected hemodynamic changes if any. A convenience sampling technique was employed to recruit adult dental patients attending the dental hospital in Imam Abdulrahman bin Faisal University. Participants were included if they were adults above 18 years, medically fit and well, seeking dental treatment in the interns' clinic in the dental hospital, and consented to participate. Patients with any medical conditions, even if controlled, pregnant women, or those taking any medications were excluded from the study.

The participants were briefed about the study aim, full details of the study procedure, and benefits of the research project both in Arabic for native speakers and in English for expatriates. They have also been informed that their participation in the study is voluntary and will not affect their treatment within the clinics. In addition to that, they were ensured that their privacy and confidentially will be maintained during the collection, analysis, and reporting of data. Written informed consent was signed by each participant and two researchers prior to the start of any procedure.

Dental anxiety was measured using the Arabic version of the Modified Dental Anxiety Scale (MDAS), a previously validated tool for measuring dental anxiety. 18 The MDAS questionnaire is a modified version composition of Corah's dental anxiety scale. 19 The questionnaire was used in many studies and documented to be quick, easy and reliable, with no instrumental effects. 19 20 21 22 The questionnaire comprises five questions about five different scenarios with responses recorded in a five-point Likert scale, where 1 refers to “not anxious” and 5 as “extremely anxious,” yielding a total score ranging from 5 to 25. 18 Patients were then categorized based on their total score into those with no dental anxiety (participants who scored less than 5), low dental anxiety (a score ranging from 5 to 10), moderate dental anxiety for those who scored between 11 to 13, high dental anxiety for scores from 14 to 19, and dental phobia for scores 19 and above.

Spot vital signs including heart rate, oxygen saturation and blood pressure were measured before, during, and after each dental procedure using an electronic sphygmomanometer (LXi device). Before dental procedure, the patient was seated in an upright position in the dental chair, the cuff of device was placed on the left arm of the patient, and the pulse oximeter sensor was placed on the index finger. The same measurement was repeated midway during the dental procedure and after the procedure was completed. Blood pressure, oxygen saturation, and heart rate were measured by one member of the research team at all three-time points. Another researcher was present to make sure that the parameters were recorded correctly and precisely for each patient.

A brief self-structured questionnaire investigating patient's demographics, oral health, and information about type of the dental procedure and its duration was filled by each participant and linked with his/her vital signs measurements and MDAS score. The data was collected on both treatment and emergency departments, where different dental procedures were performed by the interns, such as extractions, restorations, and scaling.

Measurement of heart rate and blood pressure were categorized into three categories: no change, increased or decreased, while records of oxygen saturation were categorized either into no change or increased. Descriptive statistics were presented in means ( ± standard deviation [SD]) and frequencies (percentages). Chi-square test was used to assess the association between the study variables. Statistical significance was set at *p* -value ˂ 0.05. Statistical Package for Social Sciences (Version 22.0) was used for the analysis.

## Results

**Fig. 1:**
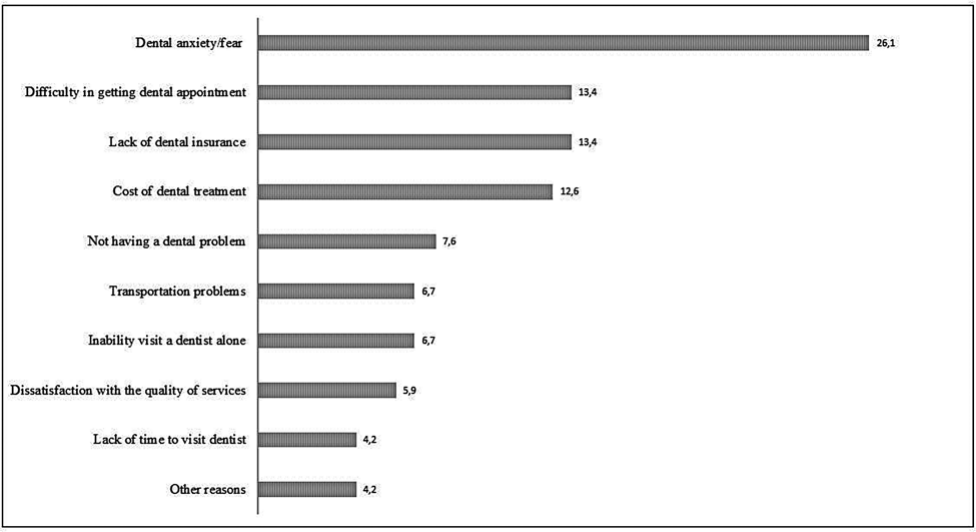
Reasons of avoiding dental visits as reported by the study participants.

A total of 119 patients participated in the study. Participants' age ranged from 18 to 59 years old with a mean ( ±  SD) of 33.7 ( ±  10.6) years. As shown in Table 1 , more than half of the participants were females (56.3%), and Saudis (58.8%). The bulk of the participants had either primary education (50.4%) or bachelor's degree (41.2%), respectively, and only half of the participants were currently employed (52.9%). Almost all participants (90.8%) brushed their teeth daily with half (51.3%) brushing twice, while only 36 participants (30.3%) perceived their oral health as good ( Table 1 ). Regarding patterns of dental visits, the majority (79.8%) did not visit the dentist regularly, with 42% visiting the dentist either for an emergency or continuing a treatment (48.7%) and half of the participants 61 (51. 3%) admitting that they tend to avoid dental treatment ( Table 1 ). Reasons for avoiding dental visits as reported by the participants are presented in ( Fig. 1 ).

**Table 1:** Background information about the study participants

Study variables	Frequency (%)
Nationality	
Saudi	70 (58.8)
Non-Saudi	49 (41.2)
Gender	
Female	67 (56.3)
Male	52 (43.7)
Education	
No formal education	3 (2.5)
Primary/secondary education	60 (50.3)
Bachelor's degree	49 (41.2)
Master's degree or above	7 (5.9)
Employment	
Employed	63 (52.9)
Not employed	56 (47.1)
Perceived oral health status	
Very poor	8 (6.7)
Poor	16 (13.4)
Fair	51 (42.9)
Good	36 (30.3)
Excellent	8 (6.7)
Daily brushing	
Yes	108 (90.8)
No	11 (9.2)
Times of daily brushing	
I don't brush daily	10 (8.4)
Once a day	29 (24.4)
Twice per day	61 (51.3)
Three or more times	19 (16)
Regular dental visits	
Yes	24 (20.2)
No	95 (79.8)
Cause of last dental visit	
Regular checkup	11(9.2)
Emergency treatment	50 (42)
Continuity of a treatment	58(48.7)
Do you avoid dental visits?	
Yes	61 (51.3)
No	58 (48.7)

Table 2 shows that restorative procedures were the most common treatment carried during the duration of the study (30%), followed by dental extractions (23.5%), while prosthodontic procedures were rarely performed (2.5%). Most of the procedures reportedly took 1 hour or less (64.7%), while the rest of the procedures took 1 hour or longer ( Table 2 ). No changes in blood pressure, heart rate, or in oxygen saturation were observed on 39.5%, 54.6%, and 97.5% of the study participants, respectively.

**Table 2:** Information about dental procedures carried and measurement of participants' vital signs

		Frequency (%)
Type of dental procedure	Restoration	36 (30.3)
	Extraction	28 (23.3)
	RCT	27 (22.7)
	Scaling with or without root planning	25 (21.0)
	Prosthodontics	3 (2.5)
Duration of the procedure	1 hour or less	77 (64.7)
	More than 1 hour	42 (35.3)
Changes in blood pressure	No change/normal	47 (39.5)
	Increase in blood pressure	57 (47.9)
	Decrease in blood pressure	15 (12.6)
Changes in heart rate	No change/normal	65 (54.6)
	Increase in heart rate	34 (28.6)
	Decrease in heart rate	20 (16.8)
Change in oxygen saturation	No change/normal	116 (97.5)
	Increased oxygen level	3 (2.5)

Abbreviation: RCT, root canal treatment.

The mean ( ±  SD) of MDAS of the study participants was 11.12 ( ±  3.9), indicative of moderate dental anxiety. Equal percent of participants had either no anxiety (21.8%) or severe anxiety (21.8%). Dental phobia was reported by only seven of the participants (5.9%). When responding to the MDAS, participants were asked about how they would feel if they had a dental appointment tomorrow, and almost half (42.9%) reported to feel slightly anxious. Sitting in the waiting area made 32.8% of the participants slightly anxious, while 37.8% mentioned they were not anxious at all. Having their teeth drilled or scaled and polished caused 14.3% and 5.9% to feel extremely anxious, respectively. On the other hand, getting a local anesthetic injection led 42% of the sample to feel slightly anxious, while 29.4% claimed to be very anxious.

**Fig. 2:**
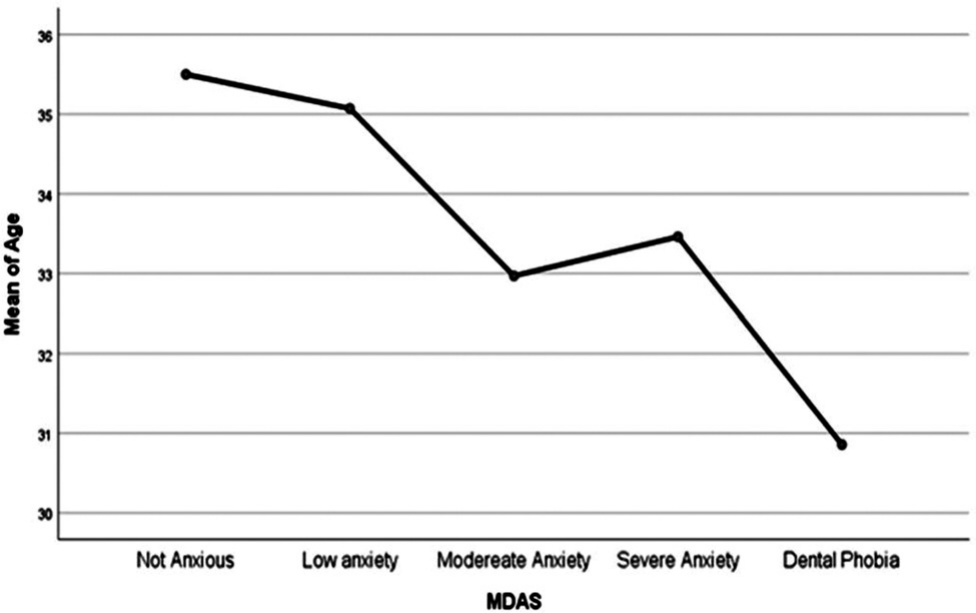
Association between age of participants and dental anxiety. MDAS, modified dental anxiety scale.

There was no significant association between MDAS scores and age ( *p* = 0.78), gender ( *p* = 0.10), nationality ( *p* = 0.56), level of education ( *p* = 0.17), or employment ( *p* = 0.48). However, it was observed that as the age increases, the severity of dental anxiety decreases ( Fig. 2 ). Although those with lower dental anxiety levels had better perceived oral health and practices, yet there was no significant association between perceived oral health, brushing habits and dental anxiety ( *p* = 0.23 and 0.13, respectively) ( Table 3 ). However, pattern of dental visits was significantly associated with MDAS scores ( *p* = 0.042), as those who visited the dentist regularly were less likely to be anxious ( Table 3 ).

**Table 3:** Association between dental anxiety and oral health practices among the study participants

Oral health practices		Dental Anxiety		*p* -Value
		Not anxious(%)	Mild anxiety(%)	
Perceived oral health status	Excellent	19.2	7.1	0.234
	Good	26.9	25	
	Fair	38.5	42.9	
	Poor	7.7	21	
Brushing frequency	I don't brush	11.5	7.1	0.134
	Once/day	26.9	25	
	Twice/day	42.3	53.6	
	More than twice/day	19.2	14.3	
Regular dental visits	Yes	34.6	21.4	0.042 a
	No	65.4	78.6	
Reason for dental visits	Checkup	3.8	7.1	0.384
	Emergency	34.6	46.4	
	Follow-up treatment	61.5	46.4	

a Statistically significant.

**Fig. 3:**
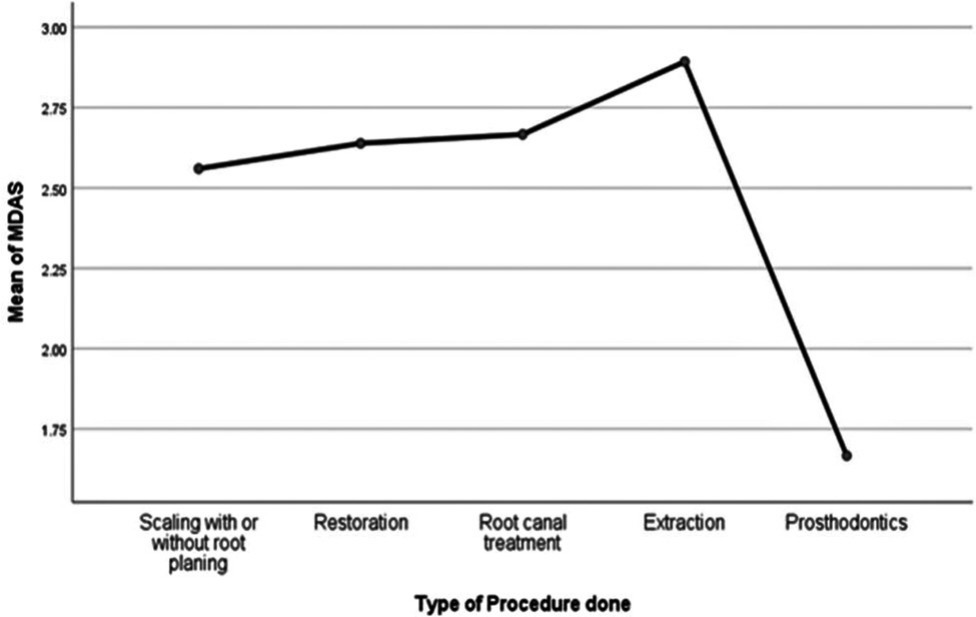
Dental anxiety levels among the study participants during different dental procedures.

Changes in vital signs were observed among the study participants within the different dental procedures ( Table 4 ). There were significant changes in blood pressure ( *p* = 0.0003) and heart rate ( *p* = 0.01) during different dental procedures, with these changes detected mainly among those undergoing root canal treatment or dental extractions. The change of oxygen saturation did not show any significant association with the type of dental procedure ( *p* = 0.33). Surprisingly, although the level of dental anxiety varied between different dental procedures, ( Fig. 3 ) however these changes were not statistically significant ( *p* = 0.52).

**Table 4:** Association between different types of dental procedures and change in the vital signs

	No change	Increased	Decreased	Total	*p* -Value
	Change of blood pressure				
Scaling	16	6	3	25	0.0003*
Restoration	18	10	8	36	
RCT	6	19	2	27	
Extraction	6	21	1	28	
Prosthodontics	1	1	1	3	
Change of heart rate					
Scaling	18	4	3	25	0.01 a
Restoration	21	6	9	36	
RCT	16	8	3	27	
Extraction	8	16	4	28	
Prosthodontics	2	0	1	3	
Change of oxygen saturation					
Scaling	24	1	0	25	0.333
Restoration	36	0	0	36	
RCT	25	2	0	27	
Extraction	28	0	0	28	
Prosthodontics	8	0	0	3	

Abbreviation: RCT, root canal treatment.

a Statistically significant.

**Fig. 4:**
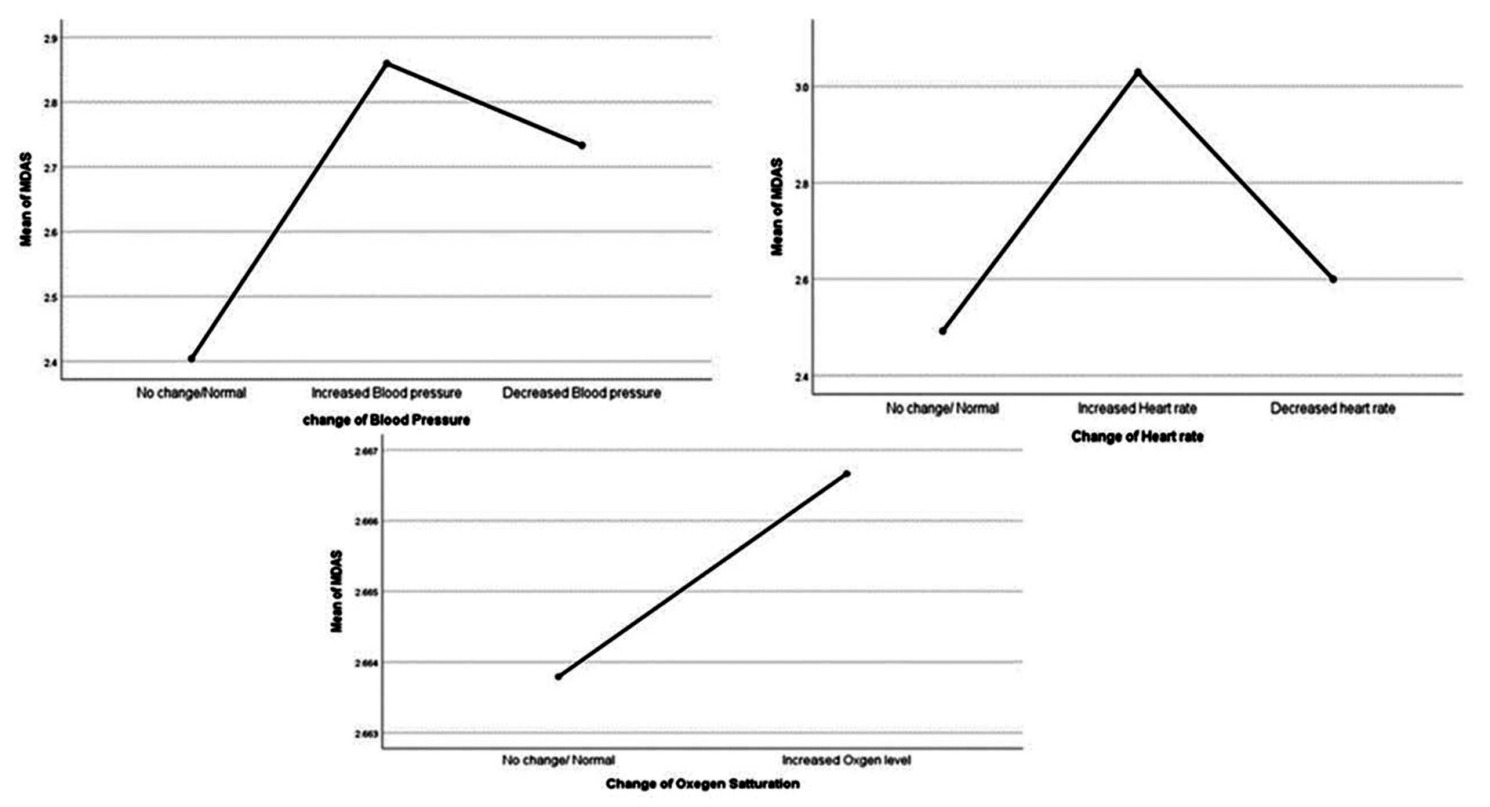
Changes in vital signs in relation to modified dental anxiety scale (MDAS) scores.

Also, changes in blood pressure, heart rate and oxygen saturation were observed in relation to mean MDAS scores ( Fig. 4 ), nevertheless no association could be established between dental anxiety and changes in blood pressure ( *p* = 0.15), heart rate ( *p* = 0.10) or oxygen saturation ( *p* = 0.99).

## Discussion

Several studies investigated dental anxiety in different contexts in an attempt to understand its predicators and assess its consequences. Dental anxiety severity, prevalence, and predicators are affected by cultural, ethnic as well as religious differences. 22 Measurement of (what can be called “universal signs”) such as heart rate, pulse rate, and blood pressure can be more accurate and objective when evaluating dental anxiety. 14 To our knowledge, this is the first study to investigate hemodynamic changes during different dental procedures and its relationship with dental anxiety in an Arab society.

In the current study, gender, age, nationality, and educational level were not associated with dental anxiety. However, many studies revealed that females, those of a younger age, and those with low educational levels are at greater risk of having dental anxiety. 6 7 10 23 24 25 26 A previously proposed biopsychosocial contextual model stated that there may be small biologically related gender differences in behaviors when it comes to emotions and anxiety; however, their expressions differ according to the context and setting. 27 The current study was conducted in a clinical setting during different dental procedures; therefore, it is expected that demographic factors, namely, gender differences would be of a lesser impact.

In previous studies, dental anxiety levels and the type of dental procedure were linked to hemodynamic changes. 28 29 30 31 32 Similar to the findings of the current study, it was observed that levels of dental anxiety can be affected by the type of dental procedure which, in turn, can cause hemodynamic changes. A fair explanation can be due to the release of corticosteroid in response to physiologic stress which, in turn, leads to hemodynamic and cardiovascular reactions. 8 This finding concedes with other studies, in that the type of dental procedure may cause different levels of dental anxiety, also in line with what was reported in many studies where oral surgeries, extractions and root canal treatments (RCT) showed significant association with dental anxiety. 5 11 33 As mentioned previously in our results, when responding to MDAS, 42% of the participants were slightly anxious and 29.4% were very anxious about dental anesthesia, in line with the findings of Gaffar et al. 11

Surprisingly, although in the current study, the level of dental anxiety also varied between different dental procedures, but these changes were not statistically significant ( *p* = 0.52), contradicting the findings of a clinical trial on 50 patients, which found that extractions ( *p* = 0 0.017) and local anesthesia injections ( *p* = 0.013) caused a tolerable rise in the systolic blood pressure. 29 Many factors that vary among individuals such as age, education, and socioeconomic and dental beliefs may lead to different reactions which, in turn, contribute to the formation dental anxiety 34 .

In the current study, it was observed that blood pressure, heart rate, and oxygen saturation differed between anxious and nonanxious patients, but these differences were not statistically significant ( *p* = 0.52). This finding is in line with what was reported by Silvestre et al, as their participants showed differences in blood pressure, heart rate, between gender, as well as between patients with and without anxiety, but these differences were also not statistically significant. 35 The type and dose of anesthetic solution injected (with or without a vasoconstrictor) as well as the type of dental procedure may explain why no significant differences in vital signs existed between anxious and nonanxious patients. 35

Changes in vital signs were also observed among the study participants within the different dental procedures. There were significant changes in blood pressure ( *p* = 0.0003) and heart rate ( *p* = 0.01) during different dental procedures, with these changes detected mainly among those undergoing RCT or dental extractions, contradicting the results of a clinical trial by Moaddabi and colleagues who observed no significant difference in diastolic blood pressure during two surgical sessions. 15 On the other hand, changes of oxygen saturation did not show any significant association with the type of dental procedure ( *p* = 0.33). Many cases of medical emergencies from fainting to death during minor dental surgical procedures have been documented in the literature; therefore, it is crucial to monitor the vital signs during dental treatment to detect these emergencies at its onset. 8

Many studies have also suggested that anxious patients tend to avoid dental visit and cancel their appointments. 19 Our results echo these findings, as it was observed that the level of dental anxiety increased proportionally with avoiding regular dentist visits ( *p* = 0.042). In the current study, about half of the participants avoided visiting the dentists, with the most common cause mentioned to be dental fear or anxiety (26.1%), followed by difficulty in getting an appointment or lack of dental insurance (13.4% each). Oyapero et al also found that delayed dental appointment influenced the levels of dental anxiety among their participants. 36 Although without a statistical significance ( *p* = 0.23 and 0.13), those with lower anxiety levels showed better perceived oral health and better practices. The relation between dental anxiety, oral health, and regular dental visits has been viewed by many as a vicious cycle, as dental fear leads to avoidance of dental visits which, in turn, aggravates the dental problems, resulting in symptom-driven treatment and more dental fear. 37 38

There are some limitations that we want to acknowledge. The study required prolonged time with the patients, multiple measurements, as well as filling the survey and signing the consent, so it was difficult to recruit the required number of participants. Many patients responded to the survey, underwent the preprocedural measurement of vital signs, and then they refused to undergo the measurement during the procedure or the post-operative one; therefore, they were not included in the study. The small sample size may have affected the results of the study. Some variables that may have influenced the changes in the vital signs such as the type of local anesthesia and the position of the chair (some procedures have been conducted with the chair on a supine position) were not recorded. Similarly, the vital signs were not monitored throughout the whole procedure, this would have allowed better evaluation of the effects of type of dental procedure as well as effect of chair position. Despite the highlighted limitations, the clinical investigation was carried by one trained single examiner, and the study used objective measurement such as vital signs and a validated tool to assess dental anxiety ensures the validity and reliability of the study.

## Conclusion

Dental anxiety is a global problem, so proper and early diagnosis cannot be overemphasized. Identifying anxious patients helps in better provision of dental care and effective managing procedures. In this study, it was found that the type of dental procedure may cause dental anxiety as well as hemodynamic changes. Therefore, close monitoring of patients with dental anxiety during the treatment is advised. And as this study was the first to be done on the region, future research should be designed to address the limitations of the current study.
